# TRANSETHNIC META-ANALYSIS OF INTERACTIONS BETWEEN GENETICS AND EARLY-LIFE SOCIOECONOMIC CONTEXT ON COGNITION

**DOI:** 10.1093/geroni/igad104.0715

**Published:** 2023-12-21

**Authors:** Jessica Faul, Minjung Kho, Wei Zhao, Kalee Rumfelt, Miao Yu, Colter Mitchell, Jennifer Smith

**Affiliations:** University of Michigan, Ann Arbor, Michigan, United States; Seoul National University, Seoul, Republic of Korea; Institute for Social Research, University of Michigan, Ann Arbor, Michigan, United States; University of Michigan, Ann Arbor, Michigan, United States; University of Michigan, Ann Arbor, Michigan, United States; University of Michigan, Ann Arbor, Michigan, United States; University of Michigan School of Public Health, Ann Arbor, Michigan, United States

## Abstract

Later-life cognitive function is influenced by genetics as well as early- and later-life socioeconomic context. However, few studies have examined the interaction between genetics and early childhood factors. Using gene-based tests (interaction sequence kernel association test [iSKAT]/iSKAT optimal unified test), we examined whether common and rare variants in 39 gene regions previously associated with cognitive performance, dementia, and related traits had an interaction with childhood socioeconomic context (parental education and financial strain) on memory performance or decline in European ancestry (EA, N = 10,468) and African ancestry (AA, N = 2,252) participants from the Health and Retirement Study. Of the 39 genes, 22 in EA and 19 in AA had nominally significant interactions with at least one childhood socioeconomic measure on memory performance and/or decline; however, only one, SLC24A4 x father’s education, remained significant after multiple testing correction (false discovery rate [FDR] < .05). In trans-ethnic meta-analysis, 2 genes interacted with childhood socioeconomic context: MS4A4A x mother’s education on memory performance, and SLC24A4 x father’s education on memory decline. Both interactions remained significant after adjusting for respondent’s own education, apolipoprotein-ε4 allele status, lifestyle factors, body mass index, and comorbidities. For both interactions in EA and AA, the genetic effect was stronger in participants with low parental education. Examination of common and rare variants in genes discovered through genome-wide association studies shows that childhood context may interact with gene regions to jointly impact later-life memory function and decline. Genetic effects may be more salient for those with lower childhood socioeconomic status.

